# Response to “Loss of Muscle Mass During Chemotherapy Is Predictive for Poor Survival of Patients With Metastatic Colorectal Cancer”

**DOI:** 10.1200/JCO.2016.68.8010

**Published:** 2016-08-01

**Authors:** Louise E. Daly, Aoife M. Ryan, Derek G. Power

**Affiliations:** Louise E. Daly and Aoife M. Ryan, University College Cork, Cork, Ireland; Derek G. Power, Cork University Hospital, Cork, Ireland

## To the Editor:

The article by Blauwhoff-Buskermolen et al^1^ highlights changes in body composition that occur during chemotherapy in patients with metastatic colorectal cancer. Literature on the impact of body composition, as defined by specific computed tomography criteria, in cancer management is evolving and, in our view, has the potential to become a valuable clinical biomarker across an array of cancers, with the ability to predict toxicity from systemic therapy as well as overall outcome.

Altered body composition is common in many cancers. Studies that have evaluated body composition changes during anticancer treatment by using single-slice computed tomography images of the abdominal region (L3) are becoming more frequent and have focused primarily on the prognostic significance of changes in muscle mass.^2,3^ The current study by Blauwhoff-Buskermolen et al^1^ focuses on the prognostic role of loss of muscle mass during chemotherapy in patients with metastatic colorectal cancer. The authors report that patients who experienced a loss of muscle mass > 9% (lowest tertile) had significantly lower survival rates than did those who experienced a loss of < 9%, which remained significant after controlling for important prognostic covariates (hazard ratio, 4.47; 95% CI, 2.21 to 9.05; *P* < .001).^1^

In the current study, low skeletal muscle index (SMI; skeletal muscle area at L3/height [m^2^]) at baseline was not associated with reduced survival, which contrasts with some,^4^ but not all, research findings.^2,5,6^ In the literature today, the most commonly used cut points for the definition of low SMI (or sarcopenia) using body composition from computed tomography scanning are those published by both Martin et al,^4^ who defined low SMI of < 43 cm^2^/m^2^ for men with body mass index < 25 cm^2^/m^2^, < 53 cm^2^/m^2^ for men with body mass index ≥ 25 cm^2^/m^2^, and < 41 cm^2^/m^2^ for women to be prognostic of reduced survival in a large cohort of 1,473 patients with lung and GI cancer; and by Prado et al,^7^ who defined low SMI of < 52.4 cm^2^/m^2^ for men and < 38.5 cm^2^/m^2^ for women as a predictor of poor overall survival in a cohort of 250 obese patients with lung and GI cancer. Both sets of cut points have been derived by using optimal stratification analysis in a North American population, and their use in other ethnicities has been questioned.^8^ This is largely in response to significant differences in skeletal muscle mass and in the rates of muscle loss with age that have been observed among different ethnic groups—African Americans, Whites, Hispanics, and Asians.^8^ Ethnic variation in cancer survival is not new, for example, East meets West in gastric cancer,^9^ and toxicity from chemotherapy can also vary depending on the population studied, for example, regional differences for the tolerability of fluoropyrimidines.^10^

Within their study, Blauwhoff-Buskermolen et al^1^ defined low SMI by using the cut points established by Martin et al.^4^ Extrapolating such cut points to a cohort of Dutch patients may have been a suboptimal approach to identify the true prevalence of low SMI and the relationship between low SMI and survival within this cohort. This may identify why changes in skeletal muscle area in this study was predictive of reduced survival, whereas low SMI at a specific time point was not. Although these North American–derived cut points have been widely applied in studies that have examined the clinical implications of low SMI, the validity of these cut points in a large European cohort has not been examined. Several of the published studies, which have reported nonsignificant relationships between low baseline SMI and reduced survival have been from European studies^2,5^ that have used the North American–derived cut points.

Further large-scale investigations are warranted in European populations, where cut points for low SMI are devised by using optimal stratification to determine its prognostic value within this population. This would provide cut points that have been validated to predict survival in large cohorts of European patients with cancer, and would not rely on cut points previously established in populations that are not representative of those being studied. In our view, the utility of body composition analysis could be even greater if ethnic variation is accounted for.
